# Incidence of intra-abdominal injuries in hemodynamically stable blunt trauma patients with a normal computed tomography scan admitted to the emergency department

**DOI:** 10.1186/s12873-024-01014-w

**Published:** 2024-06-21

**Authors:** Virginia Zarama, Nicolás Torres, Esteban Duque, Juan Pablo Arango-Ibañez, Karina Duran, Valeria Azcárate, Duban A. Maya, Álvaro I. Sánchez

**Affiliations:** 1 Facultad de Ciencias de la Salud, Universidad Icesi, Carrera 98 # 18-49, Cali, 760032 Colombia; 2 Department of Emergency Medicine, Fundación Valle del Lili, Cali, Colombia; 3 Department of Surgery, Fundación Valle del Lili, Cali, Colombia; 4 Centro de Investigaciones Clínicas (CIC), Fundación Valle del Lili, Cali, Colombia

**Keywords:** Blunt abdominal trauma, Intra-abdominal injury, High-energy trauma, Computed tomography scan

## Abstract

**Objectives:**

Blunt abdominal trauma is a common cause of emergency department admission. Computed tomography (CT) scanning is the gold standard method for identifying intra-abdominal injuries in patients experiencing blunt trauma, especially those with high-energy trauma. Although the diagnostic accuracy of this imaging technique is very high, patient admission and prolonged observation protocols are still common practices worldwide. We aimed to evaluate the incidence of intra-abdominal injury in hemodynamically stable patients with high-energy blunt trauma and a normal abdominal CT scan at a Level-1 Trauma Center in Colombia, South America, to assess the relevance of a prolonged observation period.

**Methods:**

We performed a retrospective study of patients admitted to the emergency department for blunt trauma between 2021 and 2022. All consecutive patients with high-energy mechanisms of trauma and a normal CT scan at admission were included. Our primary outcomes were the incidence of intra-abdominal injury identified during a 24-hour observation period or hospital stay, ICU admission, and death.

**Results:**

We included 480 patients who met the inclusion criteria. The median age was 33 (IQR 25.5, 47), and 74.2% were male. The most common mechanisms of injury were motor vehicle accidents (64.2%), falls from height (26%), and falls from bikes (3.1%). A total of 99.2% of patients had a Revised Trauma Score of 8. Only 1 patient (0.2%) (95% CI: 0.01–1.16) presented with an abdominal injury during the observation period. No ICU admissions or deaths were reported.

**Conclusion:**

The incidence of intra-abdominal injury in patients with hemodynamically stable blunt trauma and a negative abdominal CT scan is extremely low, and prolonged observation may not be justified in these patients.

**Supplementary Information:**

The online version contains supplementary material available at 10.1186/s12873-024-01014-w.

## Background

Blunt abdominal trauma (BAT) is a common cause of admission to the emergency department and represents a significant challenge to clinicians due to the need for a prompt diagnosis of potential intra-abdominal injuries (IAIs) to improve patient outcomes [1]. Intra-abdominal injuries have been reported in approximately 5–13% of patients with blunt trauma [2, 3], and a delayed diagnosis is associated with increased morbidity and mortality [4–6]. 

Multidetector computed tomography (CT) has become the gold standard for detecting intra-abdominal injuries in patients with blunt trauma, especially if hemodynamically stable, due to its high diagnostic accuracy [3, 7], with reported sensitivities of 75-92%, specificities of 92–100% for small bowel and mesenteric injury [7–13], and negative predictive values close to 99%^11^. However, IAI can still be present in patients with a negative CT scan, and clinical follow-up remains an important component of the diagnostic strategy in these patients.

Observation protocols for hemodynamically stable patients with suspected BAT vary among institutions and are currently based on local standards [14–18], mainly because of the scarcity of high-quality evidence supporting a specific observation time period. Severe solid organ injuries, such as hepatic, splenic, or renal lacerations, usually present symptoms within the first hour after admission [19], but other intra-abdominal injuries may present with few clinical signs for early identification [18, 19]. Nevertheless, these injuries are commonly identified within the first hours of clinical observation [17, 19], and the use of prolonged observation periods for stable patients with normal CT scans has been challenged by several studies [17, 19, 20], rendering them unnecessary and possibly increasing health-related costs and emergency department crowding. Additionally, many of these studies were conducted before the availability of multislice CT data, and all of them were conducted in the United States, with no available information on the South American population.

This study aimed to assess the incidence of intra-abdominal injuries in hemodynamically stable patients with blunt trauma and a negative abdominal CT scan admitted for observation to the emergency department at a Level-1 Trauma University Hospital in Colombia, South America.

## Methods

### Study design and setting

This retrospective observational study included patients admitted to the Emergency Department of Fundación Valle del Lili University Hospital, a Level-1 Trauma Center in Cali, Colombia, with high-energy blunt trauma and negative abdominal CT scans from January 1, 2021, to December 31, 2022. The study received approval from the Institutional Review Board under Protocol No. 2085. Informed consent was deemed unnecessary according to the study design under national regulations.

### Definitions and institutional protocols

The hospital’s current protocol mandates a 24-hour observation protocol for patients with high-energy blunt trauma and a negative abdominal CT scan, which is consistent with previous local protocols [21, 22]. The institutional imaging protocol for high-energy blunt trauma patients consists of a whole-body CT scan that includes a non-contrast brain CT scanning followed by computed tomography angiography of the neck, thorax and abdomen, including arterial and venous phase, performed in a 180 or 320 multislice CT scanner. At our institution, there is 24-hour radiologist availability to interpret all images from trauma protocol. Initially, patients underwent 12 h of observation without oral intake, followed by a 12-hour period of progressive introduction of semisolid and solid food. A complete blood count and renal function tests are usually ordered for blunt trauma patients observation, but are not mandatory as per institutional protocol. If no complications are observed at the end of this 24-hour period, the patient can be discharged home. The presence of any intra-abdominal injury identified during the 24-hour observation period or during the hospital stay by repeated imaging or surgical findings after the initial negative abdominal CT scan was defined as an event. The institutional 24-hour observation protocol flowchart for patients with blunt trauma is described in Supplementary Fig. 1.

High-energy trauma was defined as motorcyclists or bicyclists traveling at speeds exceeding 35 km/H, car speeds over 65 km/H, falls from heights of 3 m or more, collisions involving pedestrians or bicyclists with any motorized vehicle, passenger ejection from a vehicle, fatalities in the same vehicle compartment, vehicle rollovers, signs of severe impact (such as seat belt marks, abdominal wall ecchymosis, or handlebar impressions), proximity to an explosion, pelvic fractures, and incidents with unidentified mechanisms, according to Advanced Trauma Life Support guidelines [23]. 

The presence of concomitant distracting injuries was also assessed and included hemothorax, pneumothorax, pelvic fracture, long bone fracture, sternal fracture, multiple costal fractures (two or more), or scapular fracture [24]. 

### Study population

All patients who met the following criteria were included: (1) were aged 14 years or older, (2) were admitted to the emergency department (ED) due to high-energy blunt trauma, and (3) had a normal abdominal computed tomography angiography. Patients requiring immediate surgical intervention or invasive procedures, those with hemodynamic instability, those with a Glasgow Coma Scale (GCS) score less than 13 at admission, those with a diagnosis of spinal cord injury, and pregnant women were excluded. At our institution, all patients aged 14 years or older with high-energy trauma are admitted to the adult trauma unit, reflecting local epidemiology and our experience.

### Data collection

The hospital database was searched for all medical records from January 1, 2021, to December 31, 2022, and patients were selected according to the inclusion and exclusion criteria. To avoid possible bias in data collection, a data extraction form was used for retrospective chart review, and a small pilot test (10 records) was conducted to provide feedback into the data extraction form. Demographic, clinical, and outcome variables were collected by four investigators (ED, JPA, KD, VA) using the institutional platform REDCap, and an analysis of the data extraction quality was conducted by evaluating a random 10.4% sample of all records and assessing interrater agreement with kappa (0.9).

The Strengthening the Reporting of Observational Studies in Epidemiology (STROBE) statement checklist is included in the electronic supplement material [25].

### Statistical analysis

Descriptive statistics were calculated for all the variables. Categorical variables are summarized as frequencies and proportions, while quantitative variables are described using measures of central tendency and variability (mean ± standard deviation for normally distributed data or median with interquartile range for non-normally distributed data). The Kolmogorov‒Smirnov test was employed to assess the normality of these variables. Statistical analysis was performed using Stata Version 18.0.

## Results

**Fig. 1:**
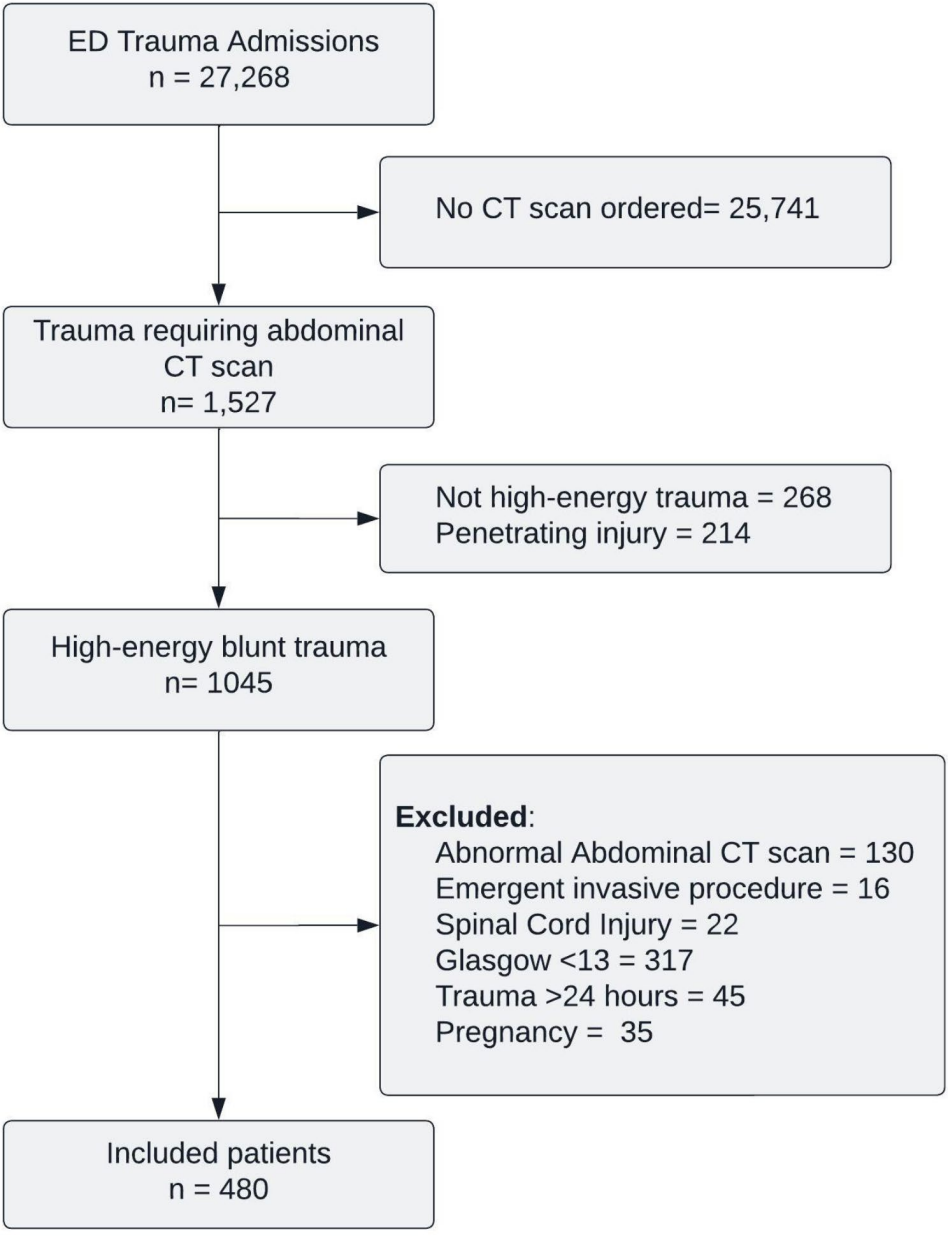
Flow diagram of patient selection. ED Emergency Department, CT Computed Tomography

From January 1, 2021, to December 31, 2022, a total of 27,268 trauma patients were admitted to the emergency department. Of these, 1,527 underwent abdominal CT scans for evaluation of abdominal trauma. A total of 482 patients were excluded due to a penetrating mechanism of injury (214) or low-energy trauma (268). Of the remaining 1,045 patients with high-energy blunt trauma, 565 were excluded based on the study’s criteria, leaving 480 patients to be included in the study (Fig. 1).

Patient baseline characteristics are summarized in Table 1. Median age of the population was 33 years (IQR = 25.5–47.0), and 74.2% of patients were male (*n* = 356). Comorbidities were present in 89 patients (18.5%), with hypertension and diabetes being the most prevalent. The most common mechanism of injury was motor vehicle accidents (64.2%), followed by falls from heights (26%). Among motor vehicle accidents, motorcycle crashes were the most prevalent (62%) followed by car accidents (19.5%). The population median Injury Severity Score (ISS) was 5 (IQR 3–6), with 12 patients having an ISS ≥ 15, and the Revised Trauma Score (RTS) was 8 points in 99.2% of the patients.

**Table 1:** Baseline characteristics

	n (%)
N	480 (100%)
Age*	33.0 (25.5, 47.0)
Male	356 (74.2%)
Comorbidities	89 (18.5%)
Hypertension	38 (7.9%)
Heart Failure	1 (0.2%)
Cerebrovascular Disease	1 (0.2%)
COPD	0 (0%)
Chronic Kidney Disease	0 (0%)
Cancer	5 (1.0%)
HIV	0 (0%)
Diabetes	12 (2.5%)
Anticoagulacion	2 (0.4%)
Mechanism of Injury	
Motor Vehicle Accident	308 (64.2%)
Fall from Heights	125 (26.0%)
Fall from Bike	15 (3.1%)
Struck by objects/others	12 (2.5%)
Other mechanism	20 (4.2%)
Motor Vehicle Accident Type	
Motorcycle Accident	191 (62.0%)
Car Accident	60 (19.5%)
Bicycle Struk	21 (6.8%)
Pedestrian Struck	32 (10.4%)
Truck accident	4 (1.3%)
Revised Trauma Score (RTS)	
8	476 (99.2%)
7	1 (0.21%)
6	3 (0.63%)
Injury Severity Score (ISS)*	5 (3,6)
Min ISS	1
Max ISS	26
Distracting injury	107 (21.5%)
Time from trauma to ED admission (hours)*	1.53 (0.98–2.72)
Vital signs at admission†	
Systolic Blood Pressure	127.7 (19.7)
Diastolic Blood Pressure	76.9 (13.0)
Mean Blood Pressure	93.4 (13.9)
Pulse Rate	85.7 (15.4)
O2Sat	98.0 (96.0–99.0)
Glasgow Score at admission*	15 (15, 15)
15	431 (89.8%)
14	41 ( 8.5%)
13	8 (1.7%)
Abdominal Pain at admission	83 (17.3%)

COPD Chronic Obstructive Pulmonary Disease, HIV Human Immunodefiency Virus, O2Sat Oxygen Saturation

* Median (Q1,Q3)

† Mean (SD)

The median time from trauma to ED admission was 1.53 h (IQR 0.98–2.72). Upon admission, the patients were hemodynamically stable, with a mean systolic arterial pressure of 127.7 mmHg (standard deviation [SD] 19.7), a mean arterial pressure of 93.4 mmHg (SD 13.9), a mean heart rate of 85.7 beats per minute (SD 15.4), and a median oxygen saturation of 98% (interquartile range [IQR] 96–99%). The majority of patients had a Glasgow score of 15 at admission (89.9%), 41 patients had a score of 14 (8.6%), and 8 patients had a score of 13 (1.6%).

Among the 480 patients, 27.3% had concomitant trauma to the head and neck, 24.2% had thoracic trauma, 33.1% had extremity trauma, 3.1% had pelvic trauma, and 8.3% had major trauma to the skin and soft tissues. Distracting injuries were identified in 107 patients (21.5% of the population): long bone fractures in 42 patients (39.3%), vertebral fractures in 21 patients (19.6%), multiple rib fractures in 18 patients (16.8%), scapular fractures in 8 patients, sternal fractures in 3 patients (2.8%), hemo-pneumothorax in 8 patients, and pelvic fractures in 3 patients (2.8%). Abdominal pain at admission was reported in 17.3% of the patients. The presence or absence of physical signs like the seat belt sign or abdominal wall hematomas was not routinely described.

The median observation time was 24.6 h (IQR 21.7–34.4 h). Notably, more than half of the patients (54.6%) had abdominal observation times longer than 24 h, which was independent of extra-abdominal injury management; 43 (9, 0%) patients were followed up longer than 48 h. Within the observation period, 5 patients developed new or increased abdominal tenderness, and 4 patients developed nausea and vomiting that required optimization of medical treatment or additional abdominal imaging, as summarized in Table 2.

**Table 2:** Observation time and changes in management

Observation Time (hours)*	24.61 (21.7, 34.4)
< 12 hours	20 (4.2%)
12–23 hours	198 (41.2%)
24–47 hours	219 (45.6%)
≥ 48 hours	43 (9.0%)
**Clinical events during observation**	
New/Increased abdominal pain	5 (1.0%)
Vomiting/Food intolerance	4 (0.8%)
New rebound tenderness	0 (%)
Tachycardia	0 (%)
Hypotension	0 (%)
Fever	0 (%)
**Change in Management**	15 (3.1%)
Exploratory Laparoscopy	1 (0.2%)
Prolong observation time	9 (1.9%)
Optimize Medical Management	2 (0.4%)
Additional Imaging studies	1 (0.2%)
Nothing by mouth	1 (0.2%)

* Median (Q1, Q3)

Only one patient (0.2%) was found to have an intra-abdominal injury, presenting with abdominal pain and rebound tenderness, despite a negative abdominal CT scan at admission. This patient, who experienced persistent abdominal pain during observation, underwent diagnostic laparoscopy 14.5 h post-admission, revealing a mesocolon hematoma without the need for further surgical interventions. Notably, no patients required admission to the intensive care unit, and no deaths were reported (Table 3).

**Table 3:** Primary outcomes in 480 patients with blunt abdominal trauma

	n (%)	95% CI	
**Intrabdominal Injury**	1 (0.2%)	0.01	1.16
**ICU admission**	0 (0%)	-	-
**Death**	0 (0%)	-	-

CI Confidence Interval, ICU Intensive Care Unit

## Discussion

In this single-center retrospective study conducted at a Level-1 Trauma Center in Cali, Colombia involving 480 consecutive patients with high-energy blunt trauma and normal abdominal CT scans admitted to the emergency department between 2021 and 2022, we identified only one patient (0.2%) with intra-abdominal injury that was not detected by initial imaging. In this patient, a mesenteric hematoma was diagnosed through laparoscopy and required no further surgical interventions. Notably, the study revealed no mortality or ICU admissions among the cohort. Following a retrospective analysis, the authors, along with a consulting expert radiologist, reexamined both the case and the images of the patient who sustained an undetected intra-abdominal injury. Despite these efforts, the initial CT scan did not reveal the lesion in the mesocolon.

The results of this study are consistent with those of previous publications in which the incidence of intra-abdominal injury in patients with blunt trauma and normal abdominal CT scans was very low. Several retrospective studies have shown that the incidence of intra-abdominal injury in patients with normal CT scans is 0% and 0.2% [8, 26–28]. Further prospective studies in children and adults have confirmed these findings [17, 20, 29, 30]. In 1998, Livingston conducted a multicenter prospective study in four Level-1 trauma centers in the United States, and from 1919 adult patients with blunt abdominal trauma and negative CT scans that were observed for 20 h or after discharge, only 4 patients were diagnosed with an IAI, for a rate of 0.2%.[18] In a single-center prospective study conducted in California by Holmes in 2012, 2734 adult patients with normal CT scans after blunt traumatic injury were followed up for at least 24 h, with an incidence of IAI reported as 0.3%. [29] In a multicenter prospective study by Kerrey et al. in 2013 in 20 emergency departments in the United States, from 3,819 children with blunt torso trauma and negative CT scans, IAIs were found in 0.4% of the population [16]. 

Finally, in a more recent single-center prospective study conducted by Benjamin et al. in 2018, which included 994 consecutive patients aged > 14 years who had negative CT scans, 9 symptomatic patients (0.9%) were diagnosed with IAIs. Notably, the study observed no such injuries in patients who were asymptomatic. This indicates that patients who experience a blunt abdominal trauma, who have negative CT scans and who do not exhibit abdominal pain or tenderness could be considered for safe discharge [17]. 

Traditionally, patients with BAT who have undergone an abdominal CT scan have been admitted for a 23-hour observation due to concerns of missed abdominal injuries [14, 15, 20]. However, prolonged observation protocols after stable blunt abdominal trauma has been challenged due to the low yield of missed diagnoses after comprehensive ED evaluation and shorter observation protocols have been proposed. Stephan et al. evaluated the usefulness of a 23-hour observation period in 4,738 blunt trauma patients over four years at a Level-1 trauma center. In this study, only 1 patient had a missed IAI that was not diagnosed by initial evaluation and CT scan, suggesting that patients with minimal injuries identified during ED evaluation and without drug or alcohol intoxication could be safely discharged home [14]. 

In another study conducted by Kendall et al., 1,169 patients with BAT and otherwise negative evaluations in the ED were admitted for observation in the ED. After a median observation time of 9.5 h, only 1 of the 1099 discharged patients was diagnosed with an IAI (splenic injury), and abdominal CT was not performed during the evaluation. The authors found that a minimum of 8 h of observation provided enough time to identify injuries in this stable cohort of patients [15]. 

Furthermore, most intra-abdominal injuries usually present early signs and symptoms. A study by Jones in 2014 found that from 3,574 blunt trauma patients admitted over a two-year period, all 285 patients diagnosed with an IAI showed signs or symptoms of injury within 8 h of admission, and all patients who ultimately required an intervention showed a sign or symptom of their injury during the first hour. The mean (SD) time to diagnosis was 74 (55) minutes, and the average observation time in this cohort was 9.5 h [19]. 

Currently, there is no consensus on the optimal observation time for hemodynamically stable with suspected BAT patients with a negative CT scan. A shorter observation time with an early discharge home in these patients could significantly decrease the costs and ED crowding for an already overwhelmed emergency health system. As early as 1996, Branney et al. reported that early discharge from the ED in this context could result in an average yearly savings of $32,874 U.S. dollars (USD) to the healthcare system [20]. In a more recent study published in 2020, Cohan et al. developed a cost-utility model in a simulated cohort of BAT patients with high risk of IAI (positive seatbelt sign) and found that ED discharge was the most cost-effective strategy with an average cost of USD $706 compared with 23-hour observation and admission compared with USD $2600 and $8,827, respectively, as long as the rate of hollow viscus injury after ED discharge is less than 2.3%.[31]

Our study, conducted at a Level-1 Trauma Center in Colombia, South America, confirms that patients with stable suspected BAT and initially negative CT scans have an extremely low incidence of IAI, and the results are consistent with similar studies from higher-income regions. These findings provide important information considering that, according to the World Health Organization, more than 90% of road fatalities occur in low-/middle-income countries, highlighting disparities in resources, access and quality of post-injury care [32]. In our study, the patient with the missed intra-abdominal injury presented with increased abdominal pain early in the course of the observation period (first hour), in accordance with previously published literature, suggesting that a short observation period of 8 h is probably adequate. Nevertheless, the safety of an early ED discharge in patients with stable suspected blunt abdominal trauma, a negative CT scan, and no abdominal pain or tenderness, provided that social support and clear follow-up instructions for consultation are assured, needs to be prospectively evaluated, as this discharge could benefit patients’ experience, ED crowding and decreased costs for the health system.

### Limitations

This study has several limitations. Given its retrospective nature, only information registered in medical records could be assessed, and the timing and presence of clinical signs and symptoms of IAI may be underestimated. The influence of extra-abdominal injuries in patients with prolonged observation times is difficult to ascertain. Clinical outcomes were only assessed through in-hospital evaluations; therefore, follow-up after ED discharge was not possible, and readmissions to other institutions for missed IAIs were not evaluated. Although this study provides data on the incidence of IAI in stable BAT patients in a South American country and confirms previously published reports from centers in the U.S., our hospital is a large university hospital in Cali, Colombia, with vast expertise in trauma patients; therefore, the findings in our center may not be generalizable to other hospitals in the region with different characteristics.

## Conclusions

The incidence of intra-abdominal injury in patients with hemodynamically stable blunt trauma and a negative abdominal CT scan is extremely low, and prolonged observation times are not justified based on the available evidence. Prospective evaluation of the safety and cost-effectiveness of early ED discharge in this context is warranted.

### Electronic supplementary material

Below is the link to the electronic supplementary material.

Supplementary Material 1

Supplementary Material 2

## Data Availability

The datasets used and/or analyzed during the current study are available from the corresponding author upon reasonable request.

## Abbreviations

BAT: Blunt abdominal trauma
COPD: Chronic obstructive pulmonary disease
CT: Computed tomography
ED: Emergency Department
HIV: Human immunodeficiency virus
IAI: Intra-abdominal injury
ICU: Intensive Care Unit
IQR: Interquartile range
SD: Standard deviation

## References

[1] Arumugam S, Al-Hassani A, El-Menyar A (2015). Frequency, causes and pattern of abdominal trauma: a 4-year descriptive analysis. J Emerg Trauma Shock.

[2] Nishijima DK, Simel DL, Wisner DH, Holmes JF (2012). Does this adult patient have a blunt intra-abdominal injury?. JAMA.

[3] Achatz G, Schwabe K, Brill S (2022). Diagnostic options for blunt abdominal trauma. Eur J Trauma Emerg Surg.

[4] Niederee MJ, Byrnes MC, Helmer SD, Smith RS (2003). Delay in diagnosis of hollow viscus injuries: effect on outcome. Am Surg.

[5] Fakhry SM, Brownstein M, Watts DD, Baker CC, Oller D (2000). Relatively short diagnostic delays (< 8 hours) produce morbidity and mortality in blunt small bowel injury: an analysis of time to operative intervention in 198 patients from a multicenter experience. J Trauma.

[6] Davis JW, Hoyt DB, McArdle MS (1992). An analysis of errors causing morbidity and mortality in a trauma system: a guide for quality improvement. J Trauma.

[7] Soto JA, Anderson SW (2012). Multidetector CT of blunt abdominal trauma. Radiology.

[8] Sherck J, Shatney C, Sensaki K, Selivanov V (1994). The accuracy of computed tomography in the diagnosis of blunt small-bowel perforation. Am J Surg.

[9] Stuhlfaut JW, Soto JA, Lucey BC (2004). Blunt Abdominal Trauma: performance of CT without oral contrast material. Radiology.

[10] Atri M, Hanson JM, Grinblat L, Brofman N, Chughtai T, Tomlinson G (2008). Surgically important bowel and/or Mesenteric Injury in Blunt Trauma: Accuracy of Multidetector CT for evaluation. Radiology.

[11] Malhotra AK, Fabian TC, Katsis SB, Gavant ML, Croce MA. Blunt Bowel and Mesenteric injuries: the role of Screening computed Tomography. J Trauma 2000;48(6). 10.1097/00005373-200006000-00001 10866242

[12] Salim A, Sangthong B, Martin M, Brown C, Plurad D, Demetriades D. Whole body imaging in Blunt Multisystem Trauma patients without obvious signs of Injury. ARCH SURG 2006;141. 10.1001/archsurg.141.5.468 16702518

[13] Fakhry SM, Watts DD, Luchette FA (2003). Current diagnostic approaches lack sensitivity in the diagnosis of Perforated Blunt Small Bowel Injury: analysis from 275,557 trauma admissions from the EAST multi-institutional HVI trial. J Trauma Inj Infect Crit Care.

[14] Stephan PJ, McCarley MC, O’Keefe GE, Minei JP (2002). 23-Hour observation solely for identification of missed injuries after trauma: is it justified?. J Trauma.

[15] Kendall JL, Kestler AM, Whitaker KT, Adkisson M-M, Haukoos JS (2011). Blunt abdominal trauma patients are at very low risk for intra-abdominal injury after emergency department observation. West J Emerg Med.

[16] Kerrey BT, Rogers AJ, Lee LK (2013). A multicenter study of the risk of intra-abdominal injury in children after normal abdominal computed tomography scan results in the emergency department. Ann Emerg Med.

[17] Benjamin E, Cho J, Recinos G (2018). Negative computed tomography can safely rule out clinically significant intra-abdominal injury in the asymptomatic patient after blunt trauma: prospective evaluation of 1193 patients. J Trauma Acute Care Surg.

[18] Livingston DH, Lavery RF, Passannante MR (1998). Admission or observation is not necessary after a negative abdominal computed tomographic scan in patients with suspected blunt abdominal trauma: results of a prospective, multi-institutional trial. J Trauma.

[19] Jones EL, Stovall RT, Jones TS (2014). Intra-abdominal injury following blunt trauma becomes clinically apparent within 9 hours. J Trauma Acute Care Surg.

[20] Brasel KJ, Borgstrom DC, Kolewe KA, Weigelt JA (1996). Abdominal computed tomography scan as a screening tool in blunt trauma. Surgery.

[21] Ferrada R, Garcia A, Cantillo E, Aristizábal G. Trauma cerrado [Internet]. In: Guías de práctica clínica basadas en la evidencia. Asociación Colombiana de facultades de medicina; 1997. http://www.medynet.com/usuarios/jraguilar/Trauma%20Abdomen.pdf.

[22] Quintero L (2015). Manejo Inicial De Los pacientes con trauma abdominal cerrado. Trauma: abordaje inicial en Los servicios de urgencias. Santiago De Cali.

[23] American College of Surgeons (2018). Abdominal and pelvic trauma. 10th Edition of the Advanced Trauma Life Support® (ATLS®) Student Course Manual.

[24] Ferrera PC, Verdile VP, Bartfield JM, Snyder HS, Salluzzo RF (1998). Injuries distracting from intraabdominal injuries after blunt trauma. Am J Emerg Med.

[25] von Elm E, Altman DG, Egger M (2007). Strengthening the reporting of Observational studies in Epidemiology (STROBE) statement: guidelines for reporting observational studies. BMJ.

[26] Fried AM, Humphries R, Schofield CN (1992). Abdominal CT scans in patients with blunt trauma: low yield in the absence of clinical findings. J Comput Assist Tomogr.

[27] Perez FG, O’Malley KF, Ross SE (1991). Evaluation of the abdomen in intoxicated patients: is computed tomography scan or peritoneal lavage always indicated?. Ann Emerg Med.

[28] Neeki MM, Hendy D, Dong F (2017). Correlating abdominal pain and intra-abdominal injury in patients with blunt abdominal trauma. Trauma Surg Acute Care Open.

[29] Holmes JF, McGahan JP, Wisner DH (2012). Rate of intra-abdominal injury after a normal abdominal computed tomographic scan in adults with blunt trauma. Am J Emerg Med.

[30] Benjamin ER, Siboni S, Haltmeier T, Lofthus A, Inaba K, Demetriades D (2015). Negative finding from computed tomography of the Abdomen after Blunt Trauma. JAMA Surg.

[31] Cohan CM, Beattie G, Tang A (2020). Does Abdominal Seat Belt sign Warrant Admission after a negative CT scan? A cost-utility analysis. J Surg Res.

[32] World Health Organization. Road traffic injuries. Fact Sheets. 13. December 2023. https://www.who.int/news-room/fact-sheets/detail/road-traffic-injuries.

