# Supplementary material for: Incidence of intra-abdominal injuries in hemodynamically stable blunt trauma patients with a normal computed tomography scan admitted to the emergency department
Source: BMC Emerg Med. 2024 Jun 21;24:103. doi: 10.1186/s12873-024-01014-w (PMC11191214; doi:10.1186/s12873-024-01014-w)
Supplement: Supplementary file 1 — Supplementary Material 1 [file 12873_2024_1014_MOESM1_ESM.docx]

**ELECTRONIC SUPPLEMENTARY MATERIAL**


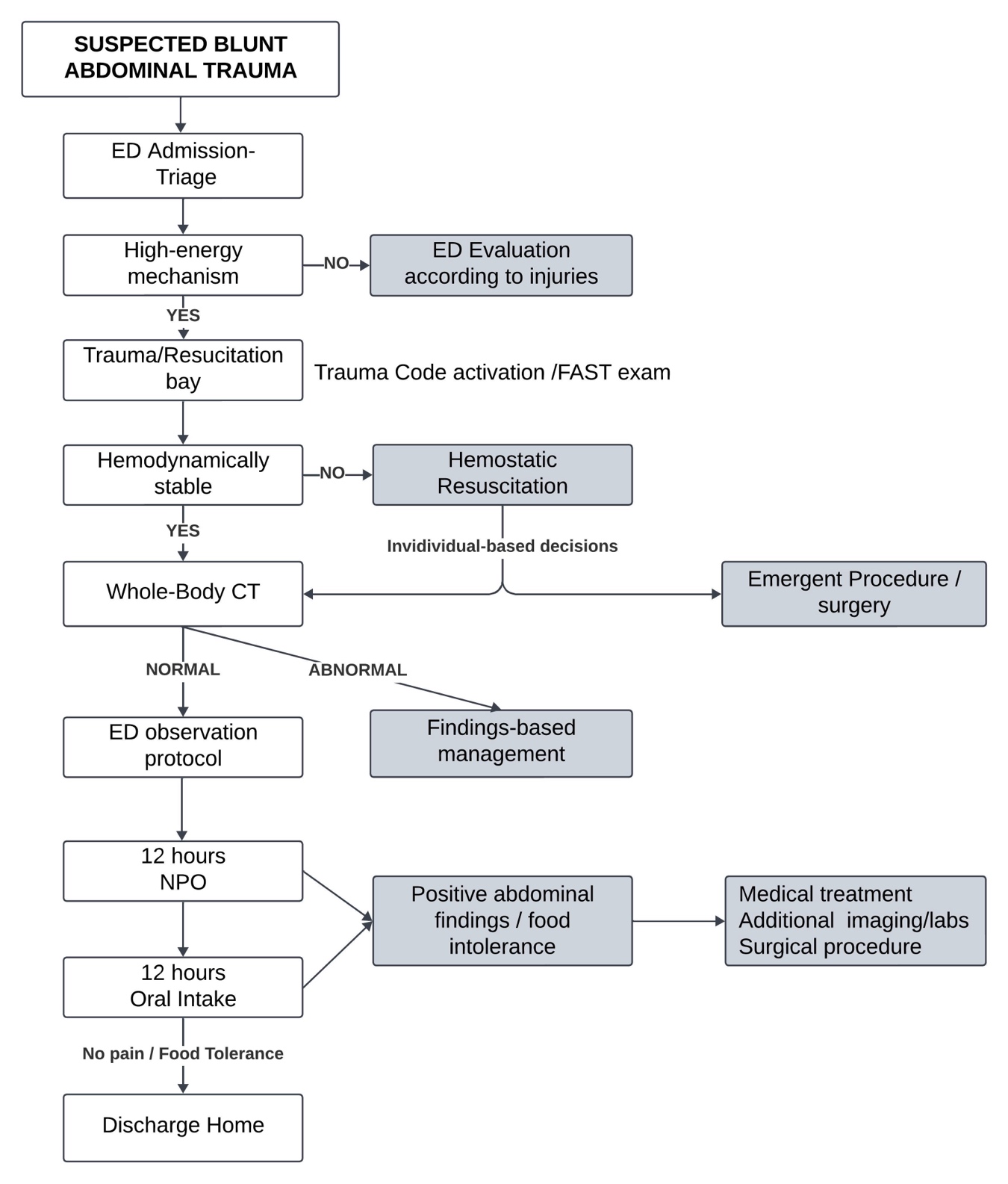


Supplementary Figure 1. Flow diagram of the institutional suspected blunt abdominal trauma protocol. ED Emergency Department, FAST Focused Assessment with Sonography for Trauma, CT Computed Tomography, NPO nothing by mouth.
